# Identification of Intermetallic Phases Present in Ti-Added Zinc Coating by Transmission Electron Microscopy

**DOI:** 10.3390/ma18174059

**Published:** 2025-08-29

**Authors:** Karolina Bracka-Kęsek, Agnieszka Bigos, Marta Janusz-Skuza, Dariusz Kopyciński

**Affiliations:** 1 Faculty of Foundry Engineering, AGH University of Krakow, al. Adama Mickiewicza 30, 30-059 Kraków, Poland; djk@agh.edu.pl; 2 Institute of Metallurgy and Materials Science, Polish Academy of Sciences, 25 Reymonta Street, 30-059 Kraków, Poland; a.bigos@imim.pl (A.B.); m.janusz@imim.pl (M.J.-S.)

**Keywords:** hot dip galvanizing process (HDG), coatings obtained in the zinc bath with addition titanium, analysis of intermetallic phase Zn-Fe-Ti, SEM/TEM characterization

## Abstract

Modeling the structure not only of whole metal products, but also of the protective coatings with which they are coated, brings a number of economic benefits through more resistant coatings and coatings that can be produced by simplifying manufacturing technology or reducing material consumption in the process. This paper presents the results of a study of dip metallization in zinc baths with Ti additions. Both steel and cast iron substrates were coated and similar results were obtained. The obtained coatings were subjected to SEM analysis with chemical composition studies, TEM characterization with selected area electron diffraction (SAED), and corrosion studies. Particle models of the elementary phases present in the zinc coating made with CaRine 3.0 software were presented and used for phase analysis. It emerged that coatings obtained in zinc baths with the addition of Ti are characterized by a more varied microstructure, the occurrence of phase separations to which Ti segregates, and higher corrosion resistance than classical zinc coatings. The higher corrosion resistance is prompted not only by the Ti content in the intermetallic phases, but also by the observed nanostructure favorably located in the alloy layer.

## 1. Introduction

Current trends in technology development focus on optimizing and increasing the quality of manufactured components. In the case of steel and cast iron, which are characterized by widespread use in many industries, it is necessary to apply protective anti-corrosion coatings due to their high propensity to corrode [1]. Dip galvanizing is an excellent solution both economically and in terms of providing corrosion protection [2]. However, it is a protection that has a lifespan of several decades depending on the process variety used and the additives that enrich the liquid zinc used in the HDG process.

**Figure 1:**
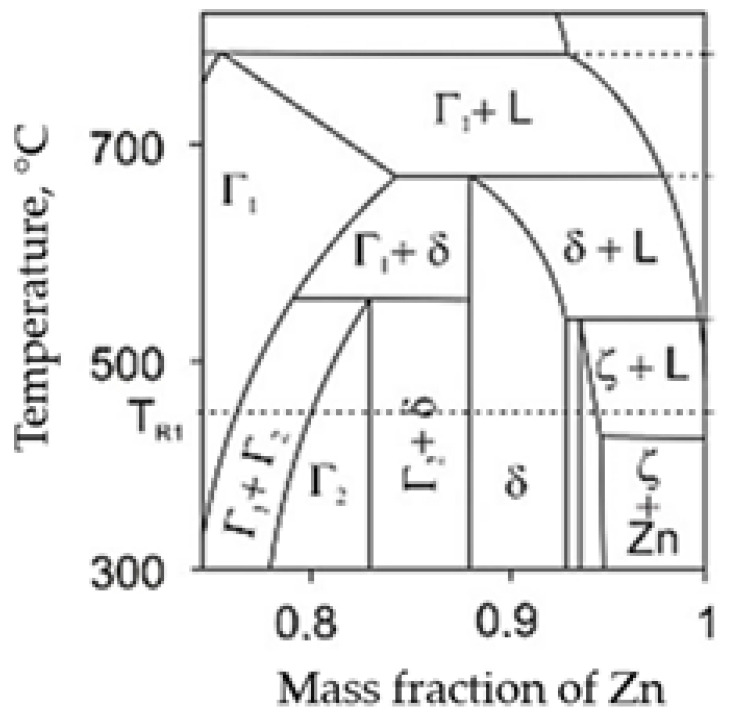
Part of the Fe-Zn phase diagram [3,4] from the zinc-rich side.

**Figure 2:**
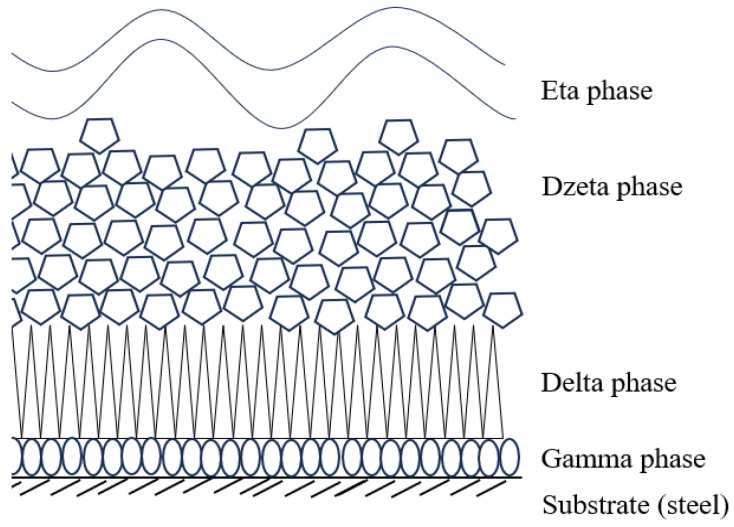
Phase Fe-Zn arrangement in the coating [19].

The corrosion protection of galvanized coatings is based on the Fe-Zn intermetallic phases formed in the coating. The alloy layer of the coating usually consists of sublayers of gamma, delta, and dzeta phases in various proportions. These phases differ in chemical composition and structure, and thus in their properties. The phase equilibrium system along with the phase designation is shown in Figure 1 [3,4]. A schematic of the coating structure is shown in Figure 2. Table 1 contains the characteristics of Fe-Zn intermetallic phases.

The shaping of zinc coatings, and consequently their properties, are affected by all the parameters of the process conducted. Researchers aim to optimize the process in all respects, such as by maximizing the resistance of coatings while reducing the cost of the process [5,6,7,8,9,10]. Numerical methods also find application in process optimization [11,12,13,14]. In order to reduce the thickness of the zinc coating, multi-component baths with synergistic effects are used [15,16,17,18].

**Table 1:** Characterization of intermetallic phases in zinc coatings [6,7].

Phase	Chemical Formula	Crystal Lattice System	Number of Atoms in an Elementary Cell
αFe	Fe	BCC	--
Γ	Fe_3_Zn_10_	BCC	52
Γ_1_	Fe_5_Zn_21_	FCC	408
δ	FeZn_10_	Hexagonal	555
ζ	FeZn_13_	Single slope layout	28
η(Zn)	Zn	HCP	6

In one study, the authors tested the addition of Ti to the zinc bath. The researchers noted the existence of new phases in such a variation of the process: Γ_2_-Fe_2_TiZn_22_ and TiZn_15_ [20]. By adding Ti to the process, the reactivity of the steel is lowered, resulting in thinner zinc coatings. However, the additional phases that are formed can deteriorate the coating’s appearance by increasing roughness. There are papers on coatings obtained by the HDG (Hot Dip Galvanizing) process with the addition of Ti [21,22,23,24,25], but none of them show the structure of the elementary cells of intermetallic phases by TEM.

**Figure 3:**
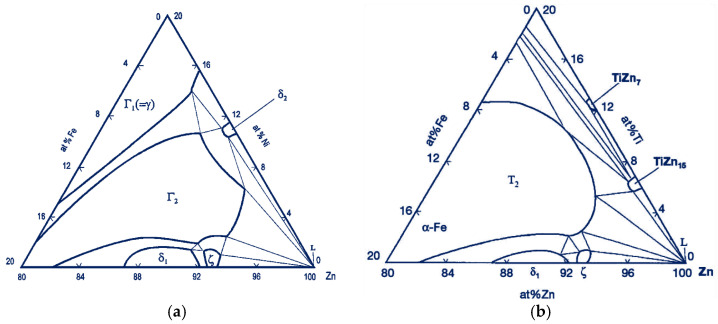
Triple phase equilibrium system at 450 °C (**a**) Fe-Zn-Ni [32]; (**b**) Fe-Zn-Ti [33].

The use of Ti additives in the zinc bath has also been aimed at limiting the growth of the zinc coating over time, so that zinc waste in the process is reduced, and the obtained coatings can be thinner. The problem of intensive zinc coating growth mainly affects alloys containing silicon and phosphorus [26,27]. Sandelin steels, where the Si content is in the <0.1% Si range, are particularly sensitive to these elements [28]. To reduce coating growth for steels in the Sandelin range, a solution was developed by adding Ni to the zinc bath. This addition has a negligible effect in forming coatings on high-silicon steels [29]. Zinc coatings with Ni obtained on Sandelin steel have a similar structure to the coating obtained on low-silicon steel [5]. An Ni content above 0.06% in the zinc bath significantly changes the equilibrium in the Fe-Zn-Ni system. The delta phase in such a variation of the process can crystallize directly from the liquid already at 450 °C [30]. In the structure of such a coating, a mixture region of δ and η phases can be distinguished. Separations of the ζ phase are formed as loose crystals at the boundary of the δ phase, without forming a continuous layer [31]. A zinc bath with Ti added, prepared for testing, can produce similar effects as the bath with Ni added, but limits the growth of the coating over the entire range of Si content. Analysis of the Fe-Zn-Ti and Fe-Zn-Ni triple systems, which show great similarity, leads to such conclusions. The systems are presented in Figure 3.

The purpose of this study was to thoroughly analyze the phases present in zinc coatings obtained in baths with varying Ti contents by transmission electron microscopy. The admixture in the tin bath can either create new phases in the coating or solutionally strengthen existing phases, depending on the amount. Usually these effects will exist simultaneously.

## 2. Materials and Methods

**Figure 4:**
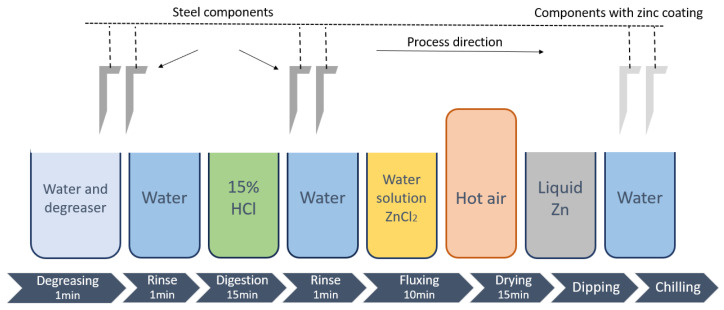
Schematic diagram of the process with the sequence of surface preparation steps.

An experimental study of immersion metallization in a zinc bath with a titanium addition was carried out. The substrate subjected to coating was steel and cast iron of EN-GJS-500-7 grade with the chemical composition shown in Table 2. The substrate to be metallized was properly prepared by pretreatment of mechanical cleaning, followed by acid etching and fluxing in an aqueous ZnCl_2_ salt solution, which was dried in a stream of boiling air to get rid of moisture from the surface. A schematic of the surface preparation is shown in Figure 4.

The experimental baths were prepared in a PTE-900 type furnace with a non-reactive ceramic crucible installed. The liquid bath zinc was obtained from pure ZnII and from recycled aircraft sheet metal. Three zinc baths were prepared with the compositions shown in Table 3. The bath without a Ti addition was the reference in the results of the obtained zinc coatings. While running the process, a similar technological problem was observed as observed for the Ni-containing baths.

Microstructural characterization in bright-field (BF) mode was performed using a TECNAI G2 SuperTWIN FEG transmission electron microscope (TEM). Phase analysis was carried out using selected area electron diffraction patterns (SADP).

Corrosion tests were performed using cyclic voltammetry in a 3.5% aqueous NaCl solution. The cyclic voltammetry method involves polarizing the test sample linearly with a time-varying potential. At the same time, the current flowing through the circuit is recorded depending on the electrode potential against the reference electrode. During the test, the working electrode is immersed in the solution together with the reference electrode and connected by an electrolytic key. An important factor in this method is the temperature at which the test is conducted, as it affects the course of the electrode reactions. For this reason, the same thermostatic conditions were maintained for all samples. The chronoamperometric curve consists of a cathodic part, lying in the range of negative potentials, and an anodic part, lying in the range of positive potentials. The change in the direction of electrode polarization occurs after the cathodic or anodic peak current. The test carried out using this method allowed the number of current peaks indicating the number of stages of the process to be determined.

## 3. Results and Discussion

### 3.1. SEM Analysis of Obtained Zinc Coatings

In order to reveal the microstructure of the obtained zinc coatings, a metalographic scan was made on the cross-section of the galvanized samples. The samples were ground and then polished until a metallographic flash was obtained. The finished smears were etched with Villel’s reagent for 1 min to highlight the interfacial boundaries in the structure, then washed in ethanol and dried.

**Figure 5:**
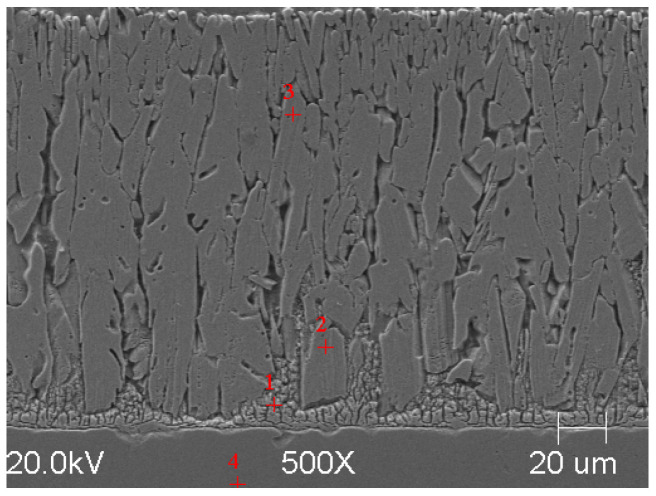
Resulting coating microstructure after 180 s immersion in Bath 0 at 450 °C.

**Figure 6:**
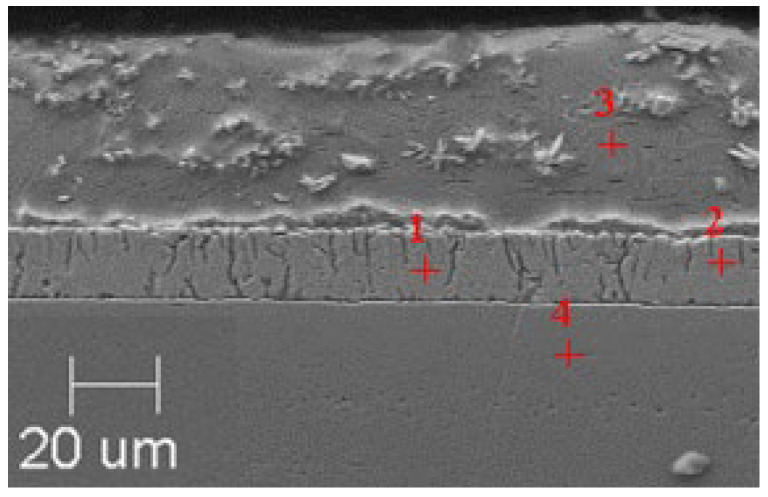
Resulting coating microstructure after 180 s immersion in Bath 0 at 550 °C.

**Figure 7:**
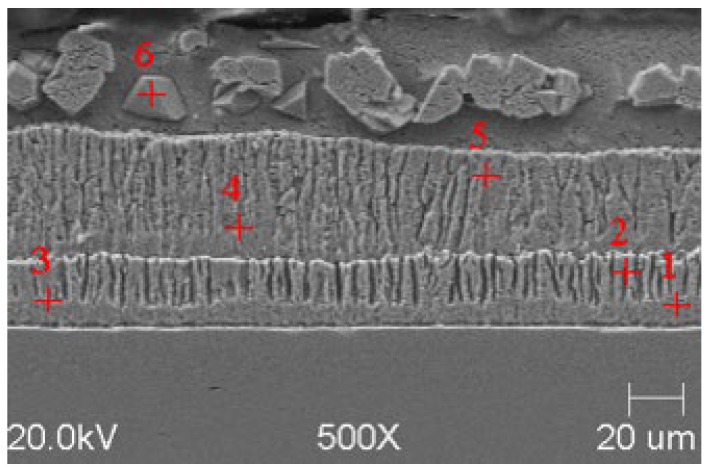
Resulting coating microstructure after 180 s immersion in Bath B at 450 °C.

**Figure 8:**
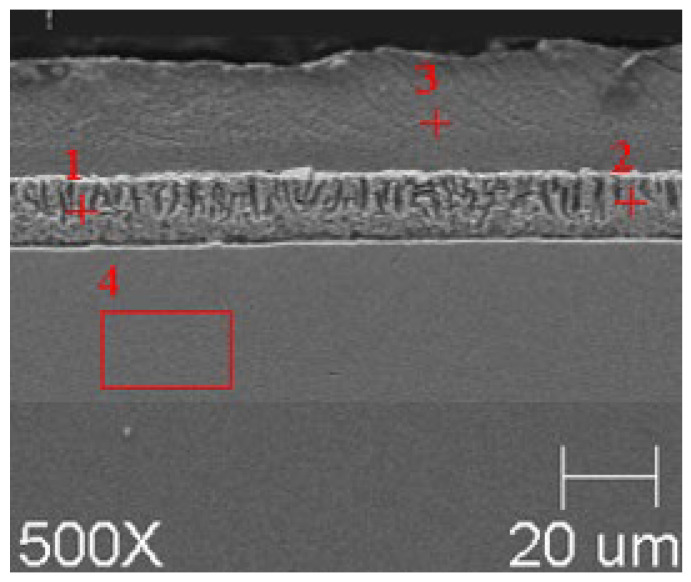
Resulting coating microstructure after 180 s immersion in Bath B at 550 °C.

**Figure 9:**
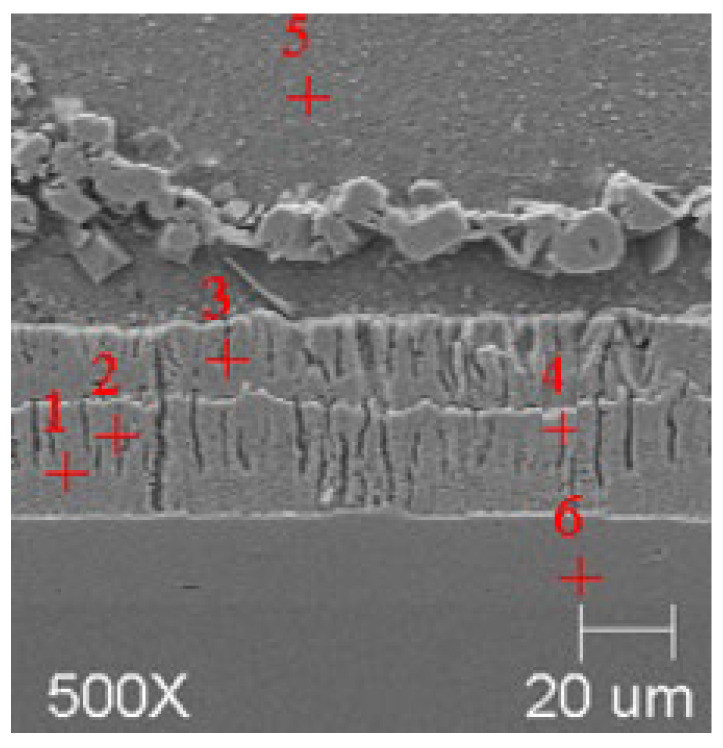
Resulting coating microstructure after 180 s immersion in Bath C at 450 °C.

**Figure 10:**
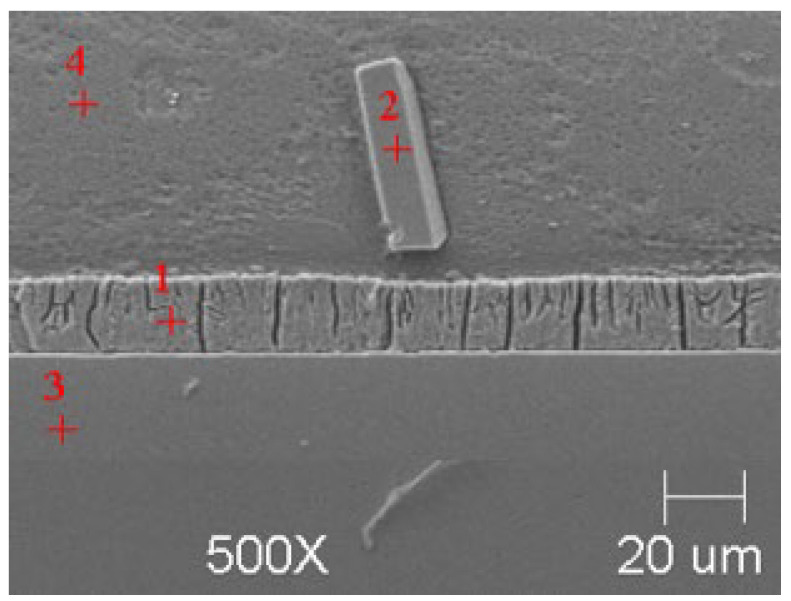
Resulting coating microstructure after 180 s immersion in Bath C at 550 °C.

SEM microanalyses were performed using a JEOL 500LV microscope (JEOL Ltd., Akishima, Japan). This made it possible to capture the microstructures of the coatings obtained in the experiment more accurately. The morphology of the resulting phases was analyzed using SEM imaging, and the chemical composition was determined using an X-ray microanalysis attachment with an EDS detector. Results of the analysis are shown in Figure 5, Figure 6, Figure 7, Figure 8, Figure 9 and Figure 10 and Table 4, Table 5, Table 6, Table 7, Table 8 and Table 9.

**Figure 11:**
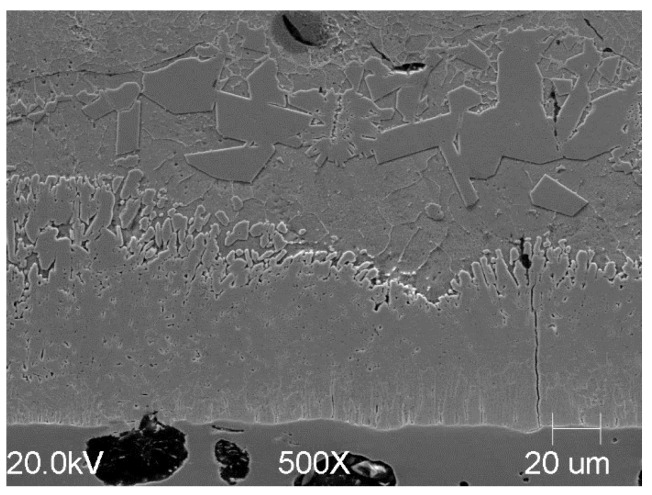
Microstructural view of the coating formed on a cast iron substrate in Bath B at 425 °C after around 300 s of immersion.

Applying the same process variation with the addition of Ti to cast iron yields similar structural effects in the coating (Figure 11). This is all the more interesting because cast iron is usually characterized by a greater coating thickness due to the composition of the substrate. In this study, a similar coating structure was obtained for both steel and cast iron. A temperature of 425 °C was used for cast iron due to experiments conducted as part of another series of studies. The photos are presented to show the versatility of the process for substrates made of various Fe-C alloys.

The results obtained from the microstructure images of zinc coatings demonstrate the importance of both the process temperature and the chemical composition of the zinc bath. A varied microstructure of coatings will also be characterized by varied anti-corrosive and mechanical properties.

Changing the chemical composition of a zinc bath by introducing a Ti additive leads to a change in the composition and structure of the coating obtained in such a bath by dip metallization. The coatings obtained in the Ti-enriched bath are characterized by separations of the phase into which Ti segregates, as confirmed by EDS analysis.

The chemical compositions of the various intermetallic phases in the coatings were similar. An enrichment of the layers with the addition of Ti was observed. No anomalies were observed in the proportion of Fe and Zn on the cross-section of the coating. The closer to the substrate, the higher the Fe content of the layer.

### 3.2. Preparation Samples of Zinc Coatings for TEM Analysis

**Figure 12:**
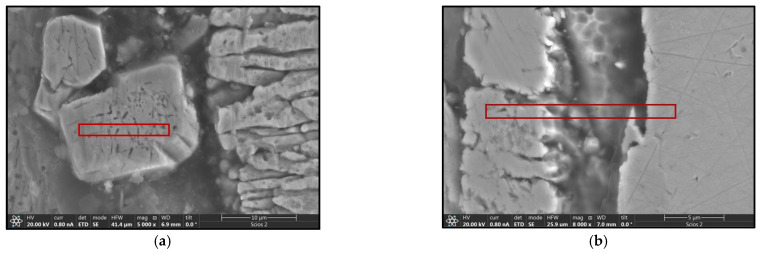

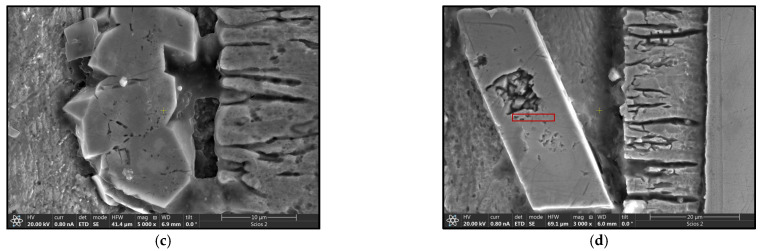
SEM images of coatings with marked areas for TEM analysis. (**a**) B450; (**b**) B550; (**c**) C450; (**d**) C550.

The use of a Tecnai G2 F20 transmission electron microscope enabled the identification, with high probability, of the phases present in the material. This analysis requires the preparation of sufficiently thin samples, the performance of which in the case of heterogeneous materials poses many difficulties. The thin foils for testing were prepared using the focused ion beam (FIB) technique on a Thermo Fisher Scientific SCIOS 2 Dual-Beam microscope due to its high accuracy and precision in cutting. Figure 12 shows the locations from which thin foils were collected for TEM observation, marked with red rectangles.

**Figure 13:**
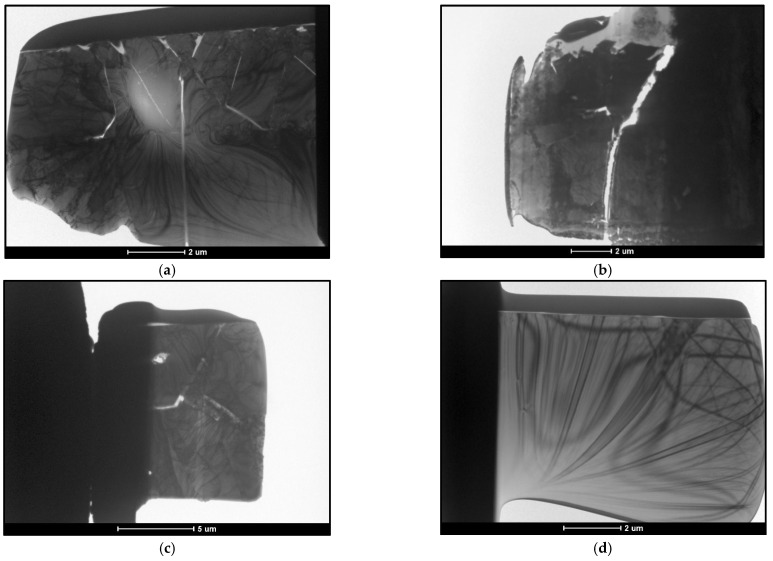
Thin foils prepared for testing TEM. (**a**) B450; (**b**) B550; (**c**) C450; (**d**) C550.

The thin foils taken for testing were 100 nm thick and approximately 10 μm × 10 μm in size. The finished preparations are shown in Figure 13. Due to the different phase composition within the sampled films, the obtained preparations were cracked.

**Figure 14:**
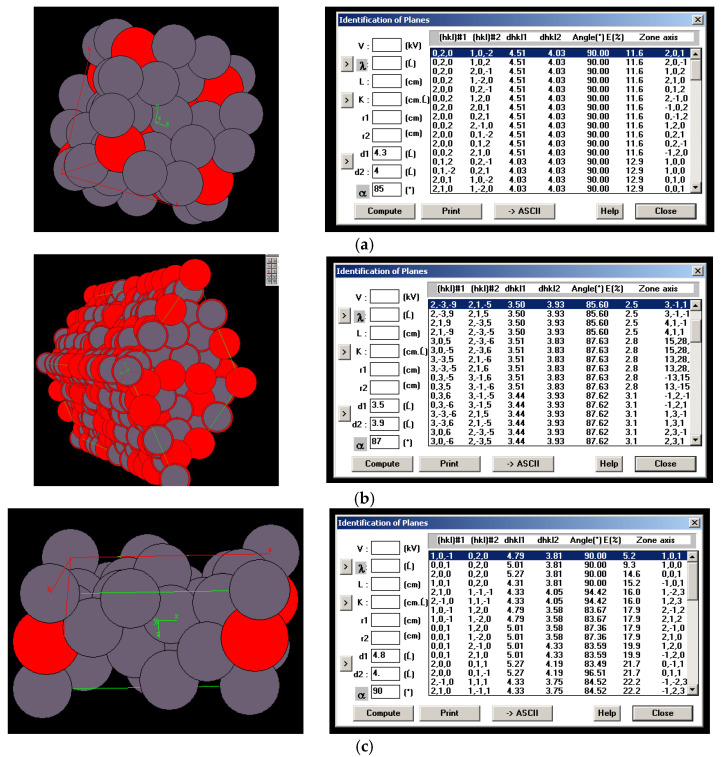
Phase particle model: (**a**) gamma; (**b**) delta; (**c**) dzeta acquired with CaRine 3.0 software.

The samples prepared in this way were subjected to TEM studies structure characterization. The result of this action can be obtained by SADP. The image of the diffraction grating was analyzed, the parameters of the lattice were determined, and the particle with the best representation was matched. The particle models of the elementary phases present in the zinc coating are shown in Figure 14.

Using CaRine 3.0 software, it is possible to construct models of phase elementary particles and determine the complexity of their structure. The largest difference in the number of atoms of an elementary cell is shown by the delta phase, where there can be even more than 500 atoms. Such large elementary particles are hard to identify unambiguously even by TEM.

### 3.3. Phase Analysis Using SADP Obtained from Prepared Samples

In the illustrations of the elementary particle models of the various phases occurring in the zinc coating, one can see a significant difference between the levels of complexity of the particle structure. The crystal structure of the elementary cell, the number of atoms, and the lattice parameters have a key effect on the properties of the phases present.

**Figure 15:**
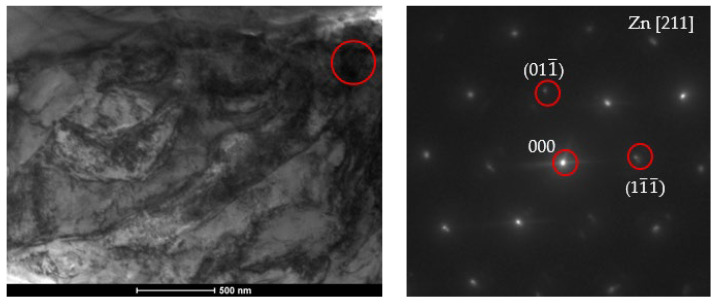
Bright field (BF) TEM image with phase analysis using SADP from region marked from the B450 sample at the site of the η (Zn) phase.

**Figure 16:**
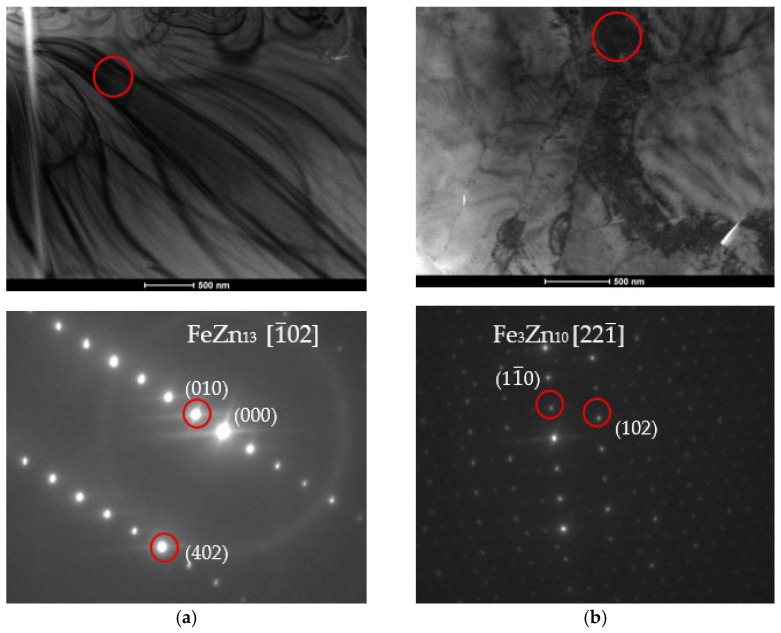

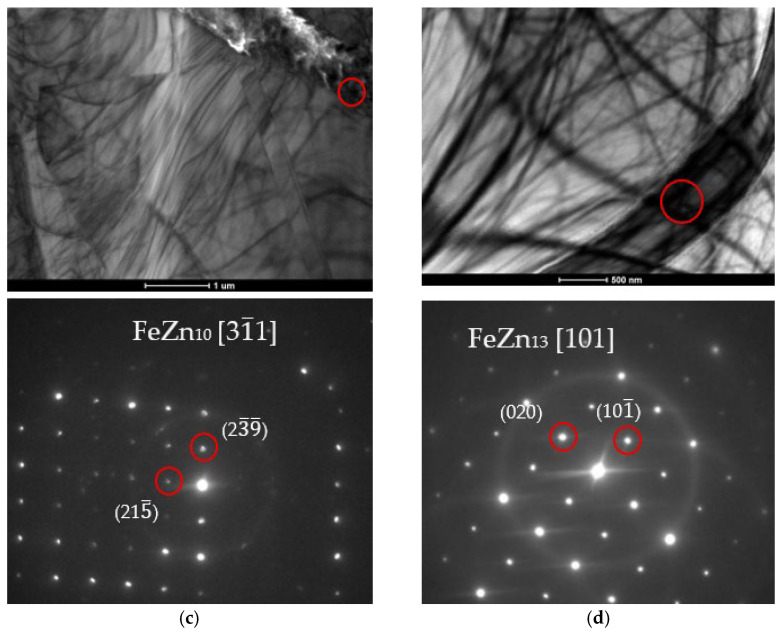
Bright field (BF) TEM images with phase analysis performed using SADP confirming the presence (**a**) FeZn_13_; (**b**) Fe_3_Zn_10_; (**c**) FeZn_10_; (**d**) FeZn_13_ phases.

The particle models should be confronted with the diffraction grids of the tested materials to determine the fit of the results to the models. The microstructure characterization of zinc coatings along with the phase analysis using SADP are presented in Figure 15 and Figure 16.

The crystal lattice parameters can be read from the photographs. The axis value is greater than three, which should disqualify the measurements. However, it is worth noting that these coefficients assume that only Zn and Fe atoms are present in the particle. In the case of the tested coatings, Ti atoms must be taken into account, as they deform and push apart the crystal lattice, as evidenced by measurements of parameters slightly above the norm.

TEM analysis allows for highly accurate determination of the type of phase being examined, along with its crystal lattice parameters. As the size of the unit cell increases, accurate analysis becomes more difficult. Intermetallic phases occurring in zinc coatings have varied chemical compositions and unit cell structures. For example, the cell of a pure zinc layer is relatively simple compared to the δ phase cell, which can have over 500 atoms in the unit cell. An additional complication in the case of the tested coatings was the addition of Ti to the zinc bath. Ti showed segregation into precipitates resembling the ζ phase in terms of structure and morphology. This phase can be considered Ti-enriched. The Ti content in these precipitates will affect both the mechanical properties of the coating and its corrosion resistance.

The authors [33] observed similar results for the tested coatings obtained by continuous galvanizing in zinc baths without the addition of Ti. The ζ and δ phases were observed and identified, while no Γ2 phase precipitates were observed. Regardless of the method used to obtain the zinc coating, the same intermetallic phases are observed, which are defined in the Fe-Zn phase equilibrium diagram. When alloying additives are used in the zinc bath, the morphology of the phases may differ, as may their proportions in the alloy layer. However, these are always the same Fe-Zn intermetallic phases [33].

The researchers conducted an experiment in which the intermetallic phases Γ1 and Γ2 were additionally obtained. The diffraction results for these phases are presented. The structure of all phases in the study revealed various types of lattice defects, which appear, among other things, as a result of non-equilibrium process conditions [34].

### 3.4. Observed Nanostructure

**Figure 17:**
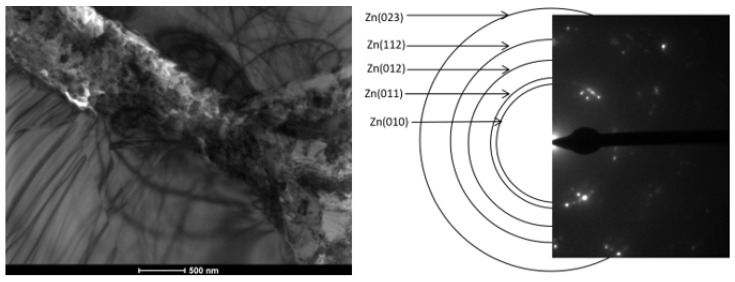
Bright field (BF) TEM image with phase analysis carried out using SADP from the place marked in BF TEM.

Nanostructural separations are of particular importance for both the mechanical and corrosion resistance of the materials obtained. The nanostructure observed in the zinc coatings studied was located between the separations of the phase to which Ti segregated. This barrier (separations between phases containing titanium + nanostructure between them) ensures increased corrosion protection in the obtained coating, as confirmed by the corrosion tests described below. The nanostructure is shown in Figure 17.

The Zn nanostructure is likely to form during rapid cooling of the coatings after metallization as a result of rapid atomic segregation. This is evidenced by the location of this structure between the Ti-enriched ζ phase precipitates.

### 3.5. Corrosion Tests of Coatings with Ti Additives

**Figure 18:**
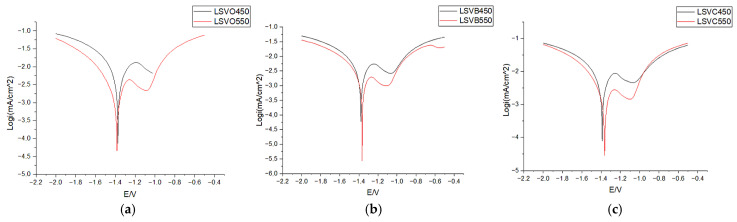
Chronovoltamperometric curves obtained in the study depending on chemical composition: (**a**) bath without additives; (**b**) B—0.05% Ti; (**c**) C—0.1% Ti.

The corrosion tests carried out confirmed the increased resistance of coatings with Ti additives compared to reference samples obtained in baths without Ti additives. The results of cyclic voltammetry are shown in Table 10 and Figure 18. Based on the results of the peak currents, it was possible to calculate the corrosion rate according to the following formula:

$$CR=\frac{k\times i_{corr}\times EW}{\rho }\;[mm/y]$$

Explanation: *k*—const. 3.27 × 10^−3^, [(mm × g)/(μA × cm × y)]

*EW*—molar mass/2, g/mol

ρ—density, g/cm^3^

*i_corr_*—peak current, [μA/cm^2^]

**Table 10:** Results of corrosion tests.

		450 °C			550 °C		
	%Ti	*E_corr_*	*i_corr_*	*CR*	*E_corr_*	*i_corr_*	*CR*
0	0	−1.373	0.013	1.95 × 10^−4^	−1.382	0.004	5.99 × 10^−5^
B	0.05	−1.3749	0.005	7.49 × 10^−5^	−1.367	0.002	2.99 × 10^−5^
C	0.1	−1.389	0.008	1.20 × 10^−4^	−1.36	0.002	2.99 × 10^−5^

The high-temperature HDG process produced coatings with greater corrosion resistance than those conducted at 450 °C. Greater corrosion resistance is associated with longer durability of steel protection by zinc coating. Increasing this resistance is an important factor in guiding the development of the technologies being designed. Such effects are achieved through process modifications (using different temperatures and reagents for preparation–treatment) as well as by adding additives to the zinc bath. In the presented studies, the additive was Ti, and it can be clearly concluded that it improves the corrosion resistance of zinc coatings. However, the concentration of Ti in the bath must be controlled, as too high a concentration leads to the formation of coatings with an unattractive appearance.

## 4. Conclusions

The TEM method makes it possible to determine the arrangement of the crystal lattice of the various precipitates present in the zinc coating. Through TEM analysis, it was possible to identify the nanostructure present in the zinc coating.

The corrosion rate of a Ti-enriched coating can be up to an order of magnitude lower compared to a pure zinc coating. This indicates a much higher corrosion resistance. Such an effect is achieved both by strengthening Fe-Zn intermetallic phases with titanium and by the presence of nanostructures.

The coating obtained by the described process is formed in a similar way on both iron and steel. However, for cast iron, this solution may be crucial in limiting the growth of the zinc coating on this type of substrate.

## Figures and Tables

**Table 2:** Chemical characteristics of the substrate used in the experiment.

	Element	C	Mn	Si	P	S	Cr	Ni	Cu	Mo	Al	Ti	Mg	V
Steel	wt.%	0.16	0.5	0.18	0.026	0.013	0.07	0.11	0.31	0.02	0.002	0.001	0.002	0.002
Cast iron		3.6	0.4	2.3	0.05	0.01	0.05	0.00	0.01	0.01	0.02	0.001	0.035	0.002

**Table 3:** Concentration of additive for zinc bath.

Bath	Addition of Titanium
0	0
B	0.05%
C	0.1%

**Table 4:** Summary of the chemical composition analysis for Bath 0 at 450 °C.

	Si	Fe	Zn
1	0.2	6.5	93.3
2	0.1	4.9	94.9
3	0.3	5.5	94.2
4	0.5	99.1	0.4

**Table 5:** Summary of the chemical composition analysis for Bath 0 at 550 °C.

	Si	Fe	Zn
1	0.4	8.2	91.5
2	0.1	7.2	92.7
3	0.3	0.1	99.7
4	0.6	98.8	0.6

**Table 6:** Summary of the chemical composition analysis for Bath B at 450 °C.

	Si	Ti	Fe	Zn
1	0.5	0	9.6	89.9
2	0.7	0.1	7.8	91.4
3	0.4	0.3	8.8	90.6
4	0.2	0.1	5.4	94.3
5	0.2	0.7	5	94.1
6	0.3	1.8	3.7	94.2
7	0.1	0.3	0.1	99.5

**Table 7:** Summary of the chemical composition analysis for Bath B at 550 °C.

	Si	Ti	Fe	Zn
1	0.4	0.5	9.2	89.9
2	0.3	0.1	10.1	89.4
3	0.3	0.1	0.6	99
4	0.4	0.1	98.8	0.7

**Table 8:** Summary of the chemical composition analysis for Bath C at 450 °C.

	Si	Ti	Fe	Zn
1	0.2	0.1	11	88.7
2	0.4	0.1	7.2	92.2
3	0.5	0.2	6.2	93.2
4	0.2	3.1	6	90.7
5	0.5	0.2	0.1	99.1
6	0.3	0.1	99.3	0.3

**Table 9:** Summary of the chemical composition analysis for Bath C at 550 °C.

	Si	Ti	Fe	Zn
1	0.1	0	8	91.8
2	0.4	3.4	6.6	89.7
3	0.2	0.1	98.9	0.8
4	0.1	0.1	0.3	99.4

## Data Availability

The original contributions presented in this study are included in the article material. Further inquiries can be directed to the corresponding author.
